# Review on the Relationship Between TyG Index and CVD Incidence in Patients With T2DM With NAFLD

**DOI:** 10.1155/ije/6707256

**Published:** 2026-07-30

**Authors:** Yuxin Zhu, Bin Yu, Haipeng Huang, Zhen Zhong

**Affiliations:** ^1^ College of Acupuncture and Massage, Changchun University of Chinese Medicine, Changchun, Jilin, China, ccucm.edu.cn; ^2^ College of Traditional Chinese Medicine, Changchun University of Chinese Medicine, Changchun, Jilin, China, ccucm.edu.cn; ^3^ Institute of Acupuncture and Tuina, Northeast Asia Academy of Traditional Chinese Medicine, Changchun University of Chinese Medicine, Changchun, Jilin, China, ccucm.edu.cn

**Keywords:** cardiovascular disease (CVD), insulin resistance (IR), review, T2DM with NAFLD, triglyceride–glucose (TyG) index

## Abstract

Type 2 diabetes mellitus (T2DM) complicated by nonalcoholic fatty liver disease (NAFLD) is a highly prevalent metabolic disorder with rapidly rising prevalence and incidence rates, and it predisposes affected individuals to multiple complications. Insulin resistance (IR) is a key pathogenic factor in patients with T2DM and concomitant NAFLD, and it is closely associated with the onset and progression of cardiovascular disease (CVD). The triglyceride–glucose (TyG) index, a novel indicator for assessing IR, has gained growing attention for its potential value in predicting CVD risk among patients with T2DM and NAFLD. Furthermore, CVD, as a common complication of T2DM with NAFLD, significantly impairs patients’ quality of life. This review aims to explore the association between the TyG index and CVD risk prediction in patients with T2DM and NAFLD and further elaborate on the underlying mechanisms. Although the exact biological mechanism linking the TyG index to CVD remains incompletely clarified, accumulating evidence has demonstrated that elevated TyG index levels are positively correlated with higher CVD prevalence. This review deepens the understanding of CVD pathogenesis in patients with T2DM and NAFLD and provides a theoretical basis for developing more effective prevention and treatment strategies.

## 1. Introduction

Quality of life is substantially impaired in individuals diagnosed with type 2 diabetes mellitus (T2DM) complicated by non‐alcoholic fatty liver disease (NAFLD), with cardiovascular disease (CVD) representing one of the most devastating adverse outcomes. The incidence of CVD is associated with multiple clinical and metabolic variables [1–5]. Accumulating evidence from comprehensive research on T2DM with NAFLD and its downstream complications has identified insulin resistance (IR) as a pivotal pathophysiological mechanism, even though the specific etiology of T2DM with NAFLD has not been fully elucidated. Currently, carotid intima‐media thickness (cIMT) represents a key surrogate marker for subclinical atherosclerosis. In obese patients with NAFLD, circulating eosinophil chemokine levels and the severity of hepatic steatosis assessed via ultrasonography can jointly predict cIMT abnormalities, which further strengthens the metabolic link between NAFLD and systemic cardiovascular risk. Among individuals with NAFLD, chronic inflammation and systemic dyslipidemia driven by excessive hepatic fat accumulation promote arterial wall thickening, a pathological alteration that can be quantitatively detected by cIMT ultrasonography. This imaging marker captures early atherosclerotic structural changes prior to plaque formation and overt cardiovascular events, indicating that hepatic metabolic dysfunction accelerates vascular aging processes. Given the shared pathophysiological pathways, including IR and oxidative stress, between NAFLD and CVD, increased cIMT in patients with NAFLD indicates a higher susceptibility to CVD [6]. These research topics have been extensively investigated in previous studies. For instance, one study [7] reported that triglyceride (TG)‐based indices exhibit superior efficacy over other conventional biomarkers in predicting cIMT thickening among young adults, particularly in obese individuals without hyperuricemia. Another population‐based cohort study enrolling children and adolescents [8] systematically evaluated the association between the triglyceride to high‐density lipoprotein cholesterol (TG/HDL‐C) ratio and early vascular morphological changes, as well as its relationship with cardiometabolic risk factors such as NAFLD. The findings confirmed that the TG/HDL‐C ratio may help identify children and adolescents at high risk of vascular structural remodeling and metabolic disorders [8]. A cross‐sectional analytical study further demonstrated that the progression of hepatic fibrosis in NAFLD patients is closely associated with aggravated IR, atherogenic dyslipidemia, and impaired hepatic synthetic function. This work further corroborated that elevated cIMT increases CVD risk in patients with NAFLD [9], suggesting that cIMT enables early screening and targeted clinical management of high‐risk individuals with concomitant T2DM and NAFLD. Multiple clinical studies [10–13] have confirmed that the presence of IR significantly increases the incidence and mortality risk of CVD in patients with T2DM [14] and NAFLD [15]. IR not only elevates individual CVD risk but also facilitates the disease progression of T2DM and NAFLD.

The concurrent presence of T2DM and NAFLD exerts synergistic detrimental effects on the disease progression and prognostic outcomes of both entities, and T2DM is independently associated with an elevated risk of hepatocellular carcinoma. Furthermore, patients with concurrent T2DM and NAFLD exhibit a significantly higher CVD risk compared with those suffering from isolated T2DM, implying a synergistic pathogenic effect that amplifies cardiovascular risk in this patient population [16].

Mechanistic studies have revealed [17] multiple signaling pathways that underlie cardiovascular sequelae and abnormal cardiac biomarkers in patients with T2DM and NAFLD. These pathways include, but are not limited to, adipose tissue dysfunction, mitochondrial impairment, gut microbiota dysbiosis, and genetic and epigenetic modifications driving IR, as well as glucotoxicity and lipotoxicity. All of the above pathological alterations collectively contribute to the development of cardiovascular complications in this clinical population and are tightly correlated with glycolipid metabolic disorders.

Accordingly, it is essential to identify reliable biomarkers that can precisely reflect IR severity and predict CVD risk in patients with T2DM with NAFLD. As an emerging metabolic marker, the triglyceride–glucose (TyG) index has attracted extensive attention due to its simplicity, cost‐effectiveness, and favorable performance in reflecting IR levels. The TyG index is calculated based on circulating TG and fasting blood glucose concentrations, correlates closely with multiple metabolic disorders, and can effectively quantify individual IR status. In patients with T2DM with NAFLD, elevated TyG index levels often indicate increased CVD susceptibility. Nonetheless, additional fundamental and clinical investigations are required to elucidate the unique impact of the TyG index in the pathogenesis of CVD secondary to T2DM and NAFLD.

This study aims to provide novel scientific evidence for the prevention and management of this intricate metabolic disorder by systematically summarizing the predictive association between the TyG index and CVD risk in patients with T2DM and NAFLD.

## 2. Review Methodology

This study is designed as a narrative review focusing on the association between the TyG index and CVD risk in patients with T2DM complicated by NAFLD. A comprehensive literature search was performed in two major electronic databases: PubMed and Web of Science. The search terms included combinations of the following key words: “triglyceride–glucose index,” “TyG index,” “type 2 diabetes mellitus,” “T2DM,” “non‐alcoholic fatty liver disease,” “NAFLD,” “cardiovascular disease,” “CVD,” “insulin resistance,” and “cardiometabolic risk.” The literature search covered all publications available up to May 2026.

Inclusion criteria were as follows: (1) human studies; (2) in vitro or animal studies; (3) full‐text articles published in English; (4) studies investigating the relationship between the TyG index and cardiovascular or metabolic outcomes in populations with T2DM, NAFLD, or both; and (5) original research articles, including cross‐sectional, cohort, and case–control studies. Exclusion criteria included (1) reviews, meta‐analyses, letters, editorials, and conference abstracts; (2) non‐English publications; and (3) studies irrelevant to the TyG index, T2DM, NAFLD, or CVD.

Two independent authors in our group screened all retrieved articles by titles and abstracts. Potentially eligible studies were then assessed through full‐text reading. Discrepancies were resolved by discussion or consultation with a third reviewer until consensus was reached. All selected studies were carefully evaluated for methodological quality and relevance to the research topic.

### 2.1. Increased Vulnerability to CVD in Patients With T2DM Combined With NAFLD

Patients with T2DM complicated by NAFLD generally experience a prolonged disease course and poorer long‐term prognosis. Individuals with both T2DM and NAFLD demonstrate a higher CVD risk compared to those with isolated T2DM or isolated NAFLD [18], suggesting a synergistic increase in future cardiovascular event risk in this comorbid population [18, 19].

Retrospective clinical investigations have shown that the incidence of CVD in patients with T2DM and NAFLD is around 1.5 to 2.5 times higher than that in the general high‐risk population [20–22]. CVD induced by ischemic heart disease, severe vascular complications, and stroke has become a leading cause of mortality among patients with T2DM and NAFLD [23–26]. Overall mortality rate attributed to CVD in this population has risen markedly, emerging as a major contributor to global years of life lost (YLL) and posing a severe public health challenge worldwide [10, 27].

### 2.2. IR as a Core Mediator of CVD Pathogenesis in T2DM With NAFLD

The probable processes of CVD generated in patients with T2DM and NAFLD are highly complex, and IR is a primary pathogenic driver that triggers numerous abnormal physiological responses and subsequently elevates the risk of CVD [28–30].

### 2.3. IR as a Key Pathological Mechanism Linking T2DM and NAFLD

IR is not only an isolated metabolic abnormal indicator but also a core pathological hub that connects the onset and progressive deterioration of T2DM and NAFLD. It constructs a pathological network of “lipid metabolism imbalance‐glucose metabolism disorder‐liver injury” through multitissue and multipathway metabolic disturbances.

IR initiates hepatic lipid metabolic disorders and lays the pathological foundation for NAFLD development. At the same time, it aggravates glucose metabolic disorder and further promotes the onset and progression of T2DM.

Accumulating research has confirmed that IR strengthens the comorbid association between T2DM and NAFLD through inflammatory and oxidative stress signaling pathways. Functionally, IR plays a bidirectional regulatory role in T2DM complicated by NAFLD, acting both as an initial pathogenic factor and a promoter of disease progression.

Numerous clinical and experimental studies have identified IR as a principal pathogenic component of global metabolic diseases and a critical mechanism underlying the co‐occurrence of T2DM and NAFLD [3].

Insulin is an essential anabolic hormone that regulates fluid balance, ion transport, and TG accumulation in adipose tissue, facilitates fatty acid esterification and storage in lipid droplets, and suppresses lipolysis. Under physiological conditions, insulin produced by pancreatic beta cells regulates glucose metabolism by promoting glucose uptake and glycogen synthesis in adipose tissue and the liver, while inhibiting hepatic glucose production. Glucagon‐like peptide‐1 (GLP‐1) inhibits hepatic gluconeogenesis and glycogenolysis by stimulating insulin secretion and suppressing glucagon release, whereas insulin itself directly inhibits hepatic gluconeogenesis, glycogenolysis, and glucose production [5]. In patients with T2DM complicated by NAFLD, reduced hepatic insulin clearance further aggravates IR and hepatic steatosis, ultimately worsening the severity of metabolic syndrome.

IR is defined as diminished cellular responsiveness to circulating insulin, which impairs the physiological regulation of blood glucose homeostasis [31]. It represents a core pathological link connecting T2DM, NAFLD, and elevated cardiovascular risk [32]. This pathological state, in which insulin‐sensitive tissues exhibit diminished responses to physiological insulin levels, significantly contributes to the onset and advancement of T2DM with NAFLD [33]. Existing research has established that IR serves a supportive and mediating role in the progression of this complex metabolic disease [1, 34]. Insulin possesses both anti‐inflammatory and proinflammatory biological properties [35]. The physiology of T2DM with NAFLD includes IR, abnormal hepatic lipid profiles, and dysregulated TG metabolism; these alterations lead to ectopic adipose tissue accumulation, impaired immune responses, and compensatory hyperinsulinemia driven by pancreatic *β*‐cell dysfunction [36]. Most circulating glucose is transported to the brain under physiological conditions, which enhances glycolysis and releases free fatty acids (FFAs) to satisfy peripheral energy demands under stress conditions [37, 38]. Pancreatic *β*‐cells mitigate IR by increasing insulin secretion, which in turn alleviates compensatory hyperinsulinemia.

Excessive caloric intake impairs insulin receptor signaling, inhibits FFA release from adipocytes, and reduces nitric oxide (NO) synthesis [39–41]. A physiological balance is normally maintained between lipid uptake (including FFA influx and de novo lipogenesis [DNL])/esterification and lipid excretion (including oxidative metabolism and lipoprotein export via very low‐density lipoprotein [VLDL]). In patients with T2DM and NAFLD, VLDL clearance capacity fails to match the accelerated rates of hepatic TG uptake and intrahepatic lipid synthesis [27]. Consequently, the immunopathogenesis of T2DM with NAFLD can be elucidated by two dominant hypotheses: Excessive dietary fat intake results in FFA overload, enhanced DNL, and diminished hepatic TG excretion; and oxidative stress, lipid peroxidation, mitochondrial dysfunction and inflammatory release jointly drive disease progression [42–44]. Consequently, IR and inflammation create a detrimental cycle that accelerates the progression of T2DM with NAFLD and other metabolic illnesses amid lipid toxicity [45]. Comparative analyses between obese and lean individuals have identified elevated IR as the primary predictor of combined T2DM and NAFLD. Additional studies have also confirmed strong correlations between serum insulin levels and hepatic ballooning degeneration as well as hepatic lobular inflammation [46–48]. The complex interaction between IR, T2DM, and NAFLD forms a detrimental cycle: Obesity induced by a high‐fat diet initiates lipotoxicity and glucotoxicity pathways, and IR further amplifies these pathological processes.

**FIGURE 1:**
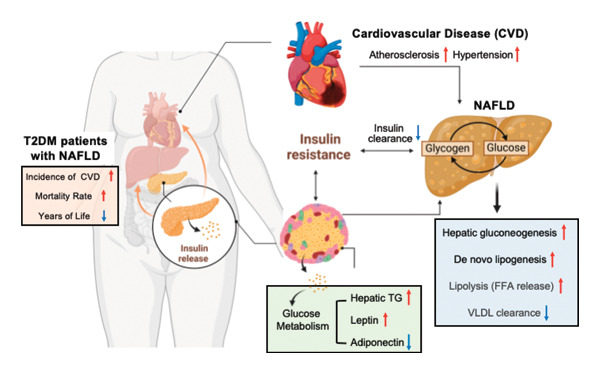
IR is a mechanism contributing to CVD in T2DM with NAFLD.

Although some scholars have argued that current evidence is insufficient to fully clarify the mechanism linking T2DM with NAFLD at this stage [1], most studies support the existence of a bidirectional association between IR and this comorbid state [4]. While establishing definitive causal relationships between IR and T2DM with NAFLD remains challenging, and the association between IR and other metabolic disorders remains ambiguous, it is not fully elucidated. As shown in Figure 1, IR is undeniably a critical pathogenic factor and core pathological component in the development of T2DM with NAFLD [2, 3].

The progression of metabolic disorders like T2DM with NAFLD inevitably leads to CVD complications such as hypertension (HTN), which exacerbates atherosclerotic lesions and increases CVD incidence and all‐cause mortality, with IR playing a central regulatory role throughout this process [49]. IR represents an independent risk factor for multiple CVD subtypes, including coronary artery disease (CAD), and it is strongly linked to elevated cardiovascular risk in patients with T2DM and NAFLD [32, 50–53].

IR is highly prevalent among patients with heart failure (HF). By impairing insulin‐mediated glucose translocation into target cells, IR induces pathological glucose and TG accumulation and disrupts systemic metabolic homeostasis [54]. Notably, the co‐occurrence of IR and TG accumulation constitutes a key driving factor in the development of metabolic syndrome—a cluster of cardiometabolic abnormalities that significantly increases the risk of adverse cardiovascular prognostic outcomes [31].

In patients with T2DM and NAFLD, intrahepatic steatosis is correlated with hepatic ceramide and diacylglycerol accumulation [55], which disrupts insulin signaling pathways and predisposes the liver to IR development. Progressive IR impairs glycemic control in patients with T2DM and NAFLD and causes pathological hyperglycemia, which further increases the risk of ischemic heart disease in this population [14]. Accordingly, patients with T2DM and NAFLD require standardized and intensive antidiabetic treatment to attain adequate blood glucose regulation [56]. Suboptimal glycemic control is closely linked to heightened CVD risk in this comorbid population. Early studies questioned the causal relationship between hyperglycemia and CVD in this population due to limited follow‐up duration, insufficient statistical robustness, and population heterogeneity; however, recent high‐quality investigations have challenged this perspective [14, 57]. Mendelian randomization‐based epidemiological studies have confirmed that genetic susceptibility to hyperglycemia is significantly correlated with elevated CVD risk in patients with T2DM and NAFLD [58]. HTN disrupts arterial wall structural integrity by promoting monocyte–macrophage endothelial adhesion, stimulating vascular smooth muscle cell proliferation, and inducing endothelial dysfunction and macrophage inflammation, thereby participating in CAD pathogenesis and increasing overall CVD risk [59–61].

Circulating leptin concentrations are markedly elevated in individuals with excess body fat and cardiometabolic diseases [62, 63]. Patients with T2DM and NAFLD present reduced adiponectin levels and elevated leptin levels, resulting in an increased leptin to adiponectin (L/A) ratio [64]. The L/A can independently forecast liver illness progression in patients with T2DM and NAFLD and is, therefore, widely recognized as a promising predictive biomarker for this comorbid condition [65]. Gremlin 1, a novel adipokine, exerts antagonistic effects on insulin signaling and correlates positively with body fat percentage and IR severity in patients with T2DM and NAFLD, making it a potential diagnostic biomarker and therapeutic target [66, 67]. Collectively, IR significantly contributes to CVD pathogenesis in patients with T2DM and NAFLD.

### 2.4. TyG Index as a Specific Biomarker for IR

The TyG index is a simple, noninsulin–based surrogate marker of IR. It is calculated using the following formula:

(1) TyG index=Lnfasting triglyceridesmg/dL×fasting glucosemg/dL/2.

Research indicates that IR not only elevates fasting blood glucose levels but is also significantly associated with heightened TyG index values [68].

As an emerging biomarker for systemic IR, the TyG index has attracted widespread research attention in the field of cardiovascular medicine. The TyG index has been validated as a reliable and easily accessible surrogate marker for IR in large population studies [31]. Current research still lacks sufficient validation data for non‐insulin–based IR indicators, including the TG/HDL‐C ratio, TyG index, and IR metabolic score (METS‐IR). An increasing number of scholars have concentrated their research on the correlation between TyG‐related variables and the occurrence of CVD.

**FIGURE 2:**
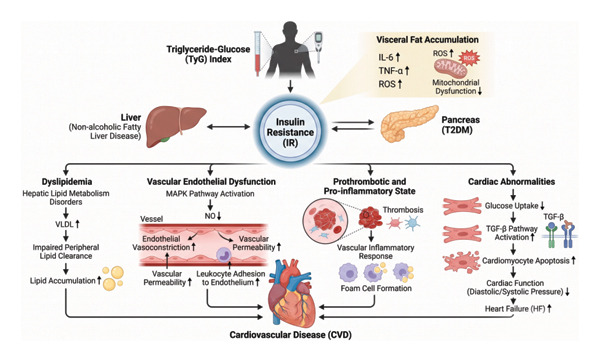
IR is a significant factor underlying CVD in T2DM with NAFLD.

The hyperinsulinemic–euglycemic clamp technique has emerged as the gold standard for assessing IR due to its high diagnostic accuracy; however, its clinical application is limited by complex operational procedures, high costs, and poor patient tolerance. Although the TyG index serves as a practical surrogate marker for IR, it exhibits relatively limited diagnostic specificity [69]. These shortcomings have restricted its large‐scale clinical application to varying degrees [70]. Calculated from fasting blood glucose and TG concentrations [71], the TyG index demonstrates superior consistency and sensitivity in IR evaluation compared with conventional surrogate markers [72], and it is significantly associated with CVD risk [31, 73]. As a critical circulating biomarker, the TyG index dynamically reflects human physiological homeostasis and health status. Its measurement sensitively captures early alterations in the internal metabolic environment and has become a routine component of general health assessments. Consensus is gradually emerging regarding the reliability and unique advantages of the TyG index as a novel IR biomarker, and it can serve as a valuable alternative indicator for IR screening and evaluation [74, 75]. In addition, as shown in Figure 2, considering the strong correlation between obesity and IR, the TYG–BMI score, which integrates the TyG index and BMI, shows better predictive performance for IR than the TyG index alone [76, 77].

### 2.5. Assessment of the TyG Index as a Predictor of CVD Risk in Individuals With T2DM With NAFLD

The correlation between the TyG index and the prevalence of CVD remains a research hotspot in cardiometabolic medicine. Patients with T2DM and NAFLD frequently present abnormal lipid profiles characterized by elevated TG‐rich lipoprotein levels [78], which highlights the necessity of exploring the correlation between the TyG index and CVD risk in this specific population.

Elevated TyG index levels are significantly associated with increased all‐cause death (ACD) risk in patients with HF (HR = 1.70, 95% CI: 1.40–2.08, *p* < 0.001). Increased TyG index also independently predicts major adverse cardiovascular events (MACEs) (HR = 2.37, 95% CI: 1.80–3.13, *p* < 0.001) and cardiovascular death (CV death) (HR = 1.63, 95% CI: 1.01–2.61, *p* < 0.001). Subgroup analysis confirms that the prognostic value of the TyG index remains stable regardless of left ventricular ejection fraction and diabetes status. Dose–response analysis has not identified a linear correlation between the TyG index and risks of ACD, MACEs, or CV death, further verifying the close association between the TyG index and long‐term prognostic outcomes in HF. Accordingly, the TyG index can be applied as a practical prognostic tool for the assessment of HF, and a high TyG index indicates elevated risks of all‐cause and cardiovascular events. Routine TyG index monitoring is therefore of great clinical significance for risk stratification and individualized management of patients with HF.

The positive association between the TyG index and HF risk has been proven [23]. Its prognostic value has also been confirmed in various CVD subtypes, including myocardial infarction [24], HTN [25], and stroke [26].

In patients with T2DM and NAFLD, elevated plasma VLDL levels induced by hepatic fat accumulation and IR further enhance cholesteryl ester transfer protein (CETP) activity. CETP mediates lipid exchange between TGs in circulating VLDL particles and cholesteryl esters in low‐density lipoprotein (LDL) and high‐density lipoprotein (HDL) particles, leading to increased TG content in HDL and LDL. These TG‐enriched lipoproteins are further hydrolyzed and remodeled by liver lipase into small, dense HDL and LDL particles [79]. Small dense LDL particles are well‐established risk factors for atherosclerosis; thus, elevated TyG levels may elevate the risk of CVD, particularly CAD, as corroborated by significant epidemiological and genetic research [27].

The TyG index and its derived indicators, including TyG–BMI, TyG–waist circumference (TyG–WC), and TyG–waist‐to‐height ratio (TyG–WHTR), have emerged as reliable IR biomarkers, with TyG‐related scores presenting an L‐shaped correlation with all‐cause mortality. Existing studies have confirmed that TyG‐related markers correlate with CVD morbidity and mortality risk in patients with T2DM and NAFLD [80, 81]. Among these derived indicators, TyG–WHTR shows the strongest predictive performance, with each unit increment associated with marked increases in all‐cause and cardiovascular mortality risk. Incorporating the TyG index or its derived parameters into CVD predictive models can improve the accuracy of mortality risk prediction in this population to a certain extent [81]. Both TyG and TyG–WC act as mediating factors linking 2‐fluorene exposure to the prevalence of overall CVD and specific cardiovascular subtypes, while TyG–BMI mediates the association between 2‐fluorene exposure and overall CVD, congestive HF, and angina pectoris [82]. A National Health and Nutrition Examination Survey (NHANES) analysis covering 1999 to 2018 demonstrated that the TyG index and its derived indicators are significant predictors of mortality in hypertensive individuals [53]. Cox regression analyses have further identified the TyG index as a major determinant of CVD mortality and verified that its inclusion can substantially improve the predictive efficiency of CVD mortality models [83]. The TyG index and its derived markers serve as valuable prognostic predictors for hypertensive patients, and their application in risk prediction models optimizes cardiovascular risk stratification and facilitates early identification of high‐risk individuals among patients with T2DM and NAFLD.

Although cross‐sectional studies have confirmed a positive correlation between the TyG index and coronary heart disease (CHD) risk as well as CVD severity in patients with T2DM and NAFLD [83, 84], large‐scale prospective cohort studies investigating the prognostic value of TyG‐related indicators for new‐onset CVD in this population remain scarce.

Two recent independent studies have reported a negative correlation between TyG index levels and all‐cause mortality [85, 86]. Therefore, updated meta‐analyses are urgently needed to integrate existing evidence, fill current knowledge gaps, and provide a more comprehensive summary of research findings in this field.

Multiple researchers have adopted the Cox proportional hazards model combined with restricted cubic splines (RCS) to evaluate the relationships between TyG‐related indicators and risks of overall CVD, CHD, stroke, all‐cause mortality, and cardiovascular mortality. Accumulating evidence consistently supports a positive association between elevated TyG index levels and adverse prognostic outcomes in HF patients [87]. This correlation is largely attributable to the fact that a high TyG index reflects severe systemic IR, which exacerbates metabolic disorders and maintains a persistent proinflammatory state, thereby accelerating HF pathogenesis.

IR induces excessive systemic inflammatory responses, a core pathological driver of cardiovascular injury. Under IR conditions, circulating levels of proinflammatory cytokines, including C‐reactive protein (CRP), tumor necrosis factor‐α (TNF‐α), and interleukin‐6 (IL‐6), are significantly upregulated. These proinflammatory mediators exert direct cytotoxic effects on cardiomyocytes, impair myocardial contractile function, and further aggravate HF progression [16, 17, 31].

IR impairs cardiovascular homeostasis through multiple pathological pathways, primarily by inducing endothelial dysfunction and increasing vascular wall permeability; these alterations promote platelet aggregation and vasoconstriction, ultimately leading to vascular dysfunction and atherosclerotic plaque formation. The resulting hemodynamic burden on the myocardium further deteriorates HF severity [31, 87, 88].

Notably, IR disrupts autonomic nervous system (ANS) homeostasis and triggers excessive sympathetic nervous system (SNS) activation. Overactive sympathetic signaling induces multiple adverse cardiac responses, including increased cardiac afterload, accelerated heart rate, and elevated myocardial oxygen consumption; these changes are particularly detrimental in HF patients with compromised myocardial reserve function. Persistent SNS overactivation also promotes myocardial fibrosis and ventricular structural remodeling, which further impairs ventricular systolic and diastolic function.

IR disrupts systemic glucose homeostasis and induces pathological hyperglycemia. IR and hyperglycemia synergistically trigger a series of metabolic disorders dominated by dyslipidemia [31, 85, 86, 89]. This dyslipidemic state is characterized by elevated circulating LDL cholesterol and TG levels alongside reduced HDL cholesterol concentrations [90], a lipid profile that accelerates atherosclerotic lesion progression and exacerbates HF pathogenesis.

At the cellular level, IR inhibits glucose utilization in cardiomyocytes and forces a metabolic shift toward fatty acid oxidation as the primary energy source. This metabolic reprogramming increases myocardial oxygen demand, which cannot be adequately compensated under HF conditions with impaired coronary perfusion. The resulting imbalance between myocardial oxygen supply and demand renders cardiomyocytes more vulnerable to ischemic injury and functional deterioration [31].

In summary, the exacerbation of HF under IR conditions is mediated by a coordinated network of pathological mechanisms, including systemic inflammation, endothelial dysfunction, ANS imbalance, and dyslipidemia. As a validated surrogate marker for IR severity, the TyG index holds important clinical value for prognostic evaluation of HF and provides critical insights into disease progression and long‐term outcomes.

**FIGURE 3:**
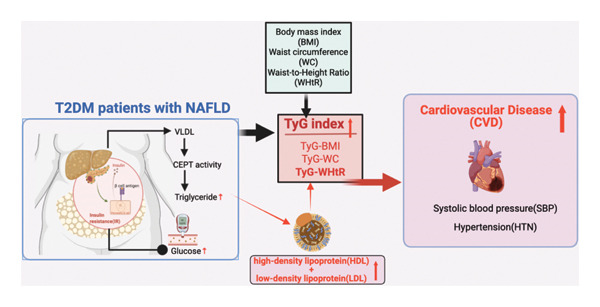
Assessment of the TyG index as a predictor of CVD risk in individuals with T2DM with NAFLD.

Restricted cubic spline analyses have revealed a nonlinear relationship between TyG‐related indicators and CVD outcomes; elevated levels of the TyG index, TyG–BMI, TyG–WC, and TyG–WHTR are independently correlated with increased CVD incidence and mortality [15]. A retrospective cohort study focusing on middle‐aged and elderly Chinese populations confirmed a strong correlation between the TyG index and CVD occurrence, particularly among middle‐aged individuals [91]. Comparative studies evaluating the predictive performance of the TyG index, TG/HDL‐C ratio, and METS‐IR for coronary artery bypass grafting (CABG) prognosis have identified the TyG index as the most powerful predictive biomarker among the three indicators [92]. Collectively, as shown in Figure 3 and Table 1, existing studies have solidly validated the robust predictive association between the TyG index and CVD risk in patients with T2DM and NAFLD.

**TABLE 1:** Summary of key studies on the TyG index and CVD risk in patients with T2DM and NAFLD.

Study	Population	Study design	Sample size	Main finding	TyG cutoff
Zhao et al., 2022 [84]	NAFLD	Cross‐sectional	424	TyG was positively associated with CHD risk and coronary atherosclerosis severity.	Not reported
Cai et al., 2025 [31]	Heart failure	Meta‐analysis	44,275	Higher TyG was related to increased MACEs and CVD death.	Not reported
Qiao et al., 2025 [15]	MASLD	Cohort	97,331	TyG‐related indices were independently associated with CVD and mortality.	Not reported

*Note:* This table summarizes the key findings of representative studies investigating the association between the TyG index and CVD risk in patients with NAFLD.

Abbreviations: CHD, coronary heart disease; CVD, cardiovascular disease; NAFLD, nonalcoholic fatty liver disease; MASLD, metabolic dysfunction‐associated steatotic liver disease; TyG, triglyceride–glucose.

## 3. Discussion

### 3.1. Clinical Utility

#### 3.1.1. Risk Stratification

The TyG index can be easily integrated into routine clinical practice to screen patients with T2DM and NAFLD at high CVD risk and guide individualized intensive management strategies, including standardized glycemic control and targeted lipid‐lowering therapy.

#### 3.1.2. Monitoring Treatment Response

Dynamic changes in the TyG index may serve as a surrogate marker for evaluating the efficacy of IR‐targeted interventions such as sodium–glucose cotransporter 2 (SGLT2) inhibitors and GLP‐1 receptor agonists. Reductions in the TyG index are closely associated with improved cardiovascular prognostic outcomes in this patient population. Although the TyG index shows good performance in reflecting IR and predicting cardiovascular risk in patients with T2DM and NAFLD, its incremental predictive value beyond conventional markers, including HbA1c, fasting glucose, TGs, HDL‐C, LDL‐C, non‐HDL‐C, ApoB, BMI, waist circumference, HOMA‐IR, liver fibrosis scores, and ASCVD risk scores, still needs further confirmation. At present, most existing studies support that TyG can provide supplementary information for risk stratification, but it is not yet possible to confirm whether it can independently replace traditional indicators.

#### 3.1.3. Clinical Nutrition Relevance

The TyG index is also closely related to clinical nutrition and lifestyle management in patients with T2DM and NAFLD. Diet quality, obesity, visceral adiposity, weight loss, physical activity, glycemic control, and lipid management all influence IR and metabolic status, thereby affecting TyG index levels. As a simple and convenient metabolic marker, the TyG index can reflect the overall status of glucose and lipid metabolism. Therefore, it may serve as a useful tool for nutritional assessment and for monitoring the effectiveness of lifestyle interventions, including dietary adjustment and physical activity. Dynamic changes in the TyG index could help evaluate the response to weight loss, glycemic control, and lipid‐lowering strategies, providing supplementary evidence for individualized nutritional and metabolic management.

### 3.2. Research Limitations

#### 3.2.1. Residual Confounding

Observational studies cannot fully exclude residual confounding by unmeasured factors like dietary habits and physical activity, which may influence the TyG index–CVD association.

#### 3.2.2. Lack of Standardization

There is no consensus on the optimal TyG index cutoff for CVD risk stratification in T2DM–NAFLD, with different studies using varying thresholds.

#### 3.2.3. Limited Data on Causal Relationships

While the TyG index is a robust predictor, interventional studies are needed to confirm whether reducing the TyG index directly lowers CVD risk.

#### 3.2.4. Limitations of Current Evidence

Several limitations regarding the existing evidence need to be acknowledged. First, residual confounding factors may exist across included studies, which could interfere with the interpretation of the association between TyG index and CVD risk. Second, the impacts of concurrent medications were not fully adjusted in many analyses, which may affect the stability of research results. Third, most studies adopted uniform fasting conditions for data collection, while the potential influence of different fasting durations has not been well explored. In addition, ethnic disparities should not be ignored: Relevant findings may not be fully generalized to all populations due to differences in genetic background, lifestyle, and metabolic characteristics among different ethnic groups. Notably, there is still no universally recognized standard cutoff value for the TyG index globally, hindering its unified clinical application. Furthermore, most available studies are observational in design, so we cannot draw definitive conclusions on causal relationships between the TyG index and adverse cardiovascular outcomes.

## 4. Conclusion

IR serves as a pivotal pathological driver in the progression toward CVD, particularly among patients with T2DM complicated by NAFLD. The TyG index, a readily accessible surrogate marker for IR, effectively reflects the severity of IR‐mediated metabolic disturbances, including glucose and TG metabolism. Progressive IR elevation is accompanied by increased TyG index values, which indirectly indicate the activation of IR‐related pathological pathways such as atherogenic dyslipidemia, vascular endothelial dysfunction, and systemic proinflammatory/prothrombotic states. Elevated TyG index levels, as a downstream indicator of unresolved IR, correlate closely with accelerated atherosclerotic progression and subsequent CVD development, establishing the “IR–TyG–CVD” cascade as a key mechanistic link in high‐risk metabolic cohorts.

Given that CVD represents a major comorbidity and leading cause of mortality in patients with T2DM and NAFLD, further investigation into the predictive value of the TyG index for cardiovascular risk stratification in this specific population is clinically imperative. Current research on the association between the TyG index and CVD prevalence in patients with T2DM and NAFLD has become a research hotspot; however, additional studies are urgently needed to quantify the strength of this association and unravel the underlying interaction mechanisms. Moreover, the precise mechanism by which the TyG index and its derived indicators predict CVD incidence in this population remains incompletely clarified and requires further in‐depth exploration. Existing relevant studies are predominantly concentrated on Western populations, with a particular concentration in the United States. This geographical limitation highlights the pressing need for expanded research in other ethnic and regional populations to enhance the generalizability of findings and address potential ethnic or regional disparities in the utility of the TyG index for CVD risk stratification.

This study synthesizes the current body of research on the association between the TyG index and CVD incidence in patients with T2DM and NAFLD. Future research will focus on exploring additional biomarkers linked to T2DM, NAFLD, and CVD incidence. The overarching goal is to provide theoretical references for clarifying the predictive correlation between the TyG index and CVD risk specifically in patients with T2DM complicated by NAFLD through more extensive and in‐depth clinical and mechanistic investigations. The TyG index serves as a promising and practical metabolic marker for patients with T2DM and NAFLD. Nevertheless, further prospective studies are still required before it can be applied as an independent predictor of cardiovascular risk in clinical practice.

## Funding

The study was supported financially by the National Natural Science Foundation of China (NSFC) Projects (Grant No. 82575241) and the Natural Science Foundation of Jilin Province (Grant No. YDZJ202301ZYTS165).

## Consent

This statement is to certify that all authors have seen and approved the manuscript being submitted, have contributed significantly to the work, attest to the validity and legitimacy of the data and their interpretation, and agree to its submission to the journal. We attest that the article is the authors’ original work, has not received prior publication, and is not under consideration for publication elsewhere. We all consent to the copyright and license agreement and have read and agreed to the editorial policies of the journal.

## Conflicts of Interest

The authors declare no conflicts of interest.

## Data Availability

Research data are not shared.
